# Vasopressin induces apoptosis but does not enhance the antiproliferative effect of dynamin 2 or PI3K/Akt inhibition in luminal A breast cancer cells

**DOI:** 10.1007/s12032-022-01889-4

**Published:** 2022-12-02

**Authors:** Samar Sami Alkafaas, Samah A. Loutfy, Thoria Diab, Mohamed Hessien

**Affiliations:** 1 Molecular Cell Biology Unit, Division of Biochemistry, Department of Chemistry, Faculty of Science, Tanta University, Tanta, 31511 Egypt; 2 Virology and Immunology Unit, Cancer Biology Department, National Cancer Institute, Cairo University, Cairo, Egypt; 3 Division of Biochemistry, Department of Chemistry, Faculty of Science, Tanta University, Tanta, 31511 Egypt; 4 Nanotechnology Research Center, British University, Cairo, Egypt

**Keywords:** Breast cancer, Dynamin 2, Dynasore, Wortmannin, Arginine vasopressin

## Abstract

Breast cancer cells abnormally express vasopressin (AVP) and its receptors. The effect of AVP is largely orchestrated through its downstream signaling and by receptor-mediated endocytosis (RME), in which Dynamin 2 (Dyn2) plays an integral role in vesicle closure. In this work, luminal A breast cancer cells were treated with AVP, and then Dynasore (DYN) was employed to inhibit Dyn2 to explore the combined effect of AVP and Dyn2 inhibition on the survival of breast cancer cells. The results revealed that DYN alone demonstrated a concentration-dependent cytotoxic effect in AVP untreated cells. Apoptosis developed in 29.7 and 30.3% of cells treated with AVP or AVP+DYN, respectively, compared to 32.5% in cells treated with Wortmannin (Wort, a selective PI3K pathway inhibitor). More apoptosis was observed when cells were treated with DYN+Wort in presence or absence of exogenous AVP. Besides, 2 or 4- fold increases in the expression of Bax and Caspase-3, were observed in cells exposed to AVP in absence or presence of DYN, respectively. This was associated with higher levels of the autophagy marker (LC3II protein). Meanwhile, the activation of Akt protein, sequentially decreased in the same pattern. Cell’s invasion decreased when they were exposed to AVP alone or combined with DYN or/and Wort. Conclusively**,** although many reports suggested the proliferative effect of AVP, the results predict the antiproliferative and antimetastatic effects of 100 nM AVP in luminal A breast cancer cells. However, the hormone did not enhance the cytotoxic effect of Dyn 2 or PI3K pathway inhibition.

**Graphical Abstract:**

Summary of the Dynamin 2 independent AVP antiproliferative effects. Breast cancer cells expresses AVP as a Prohormone (A). At high dose of AVP, the hormone is liganded with AVP receptor (B) to initiate RME, where the endosomed complex (C) is degraded through the endosome-lysosome system, as a part of signal management. These events consume soluble Dyn2 in neck closure and vesicle fission (D). This makes the cells more substitutable to the direct apoptotic effect of DYN (E). Alternatively, at lower AVP doses the liganded AVP may initiate cAMP-mediated downstream signaling (F) and cellular proliferation. In parallel, Wort inhibits PIP2-PIP3 conversion (G) and the subsequent inhibition of PI3K/Akt/mTOR pathway leading to cell death. 
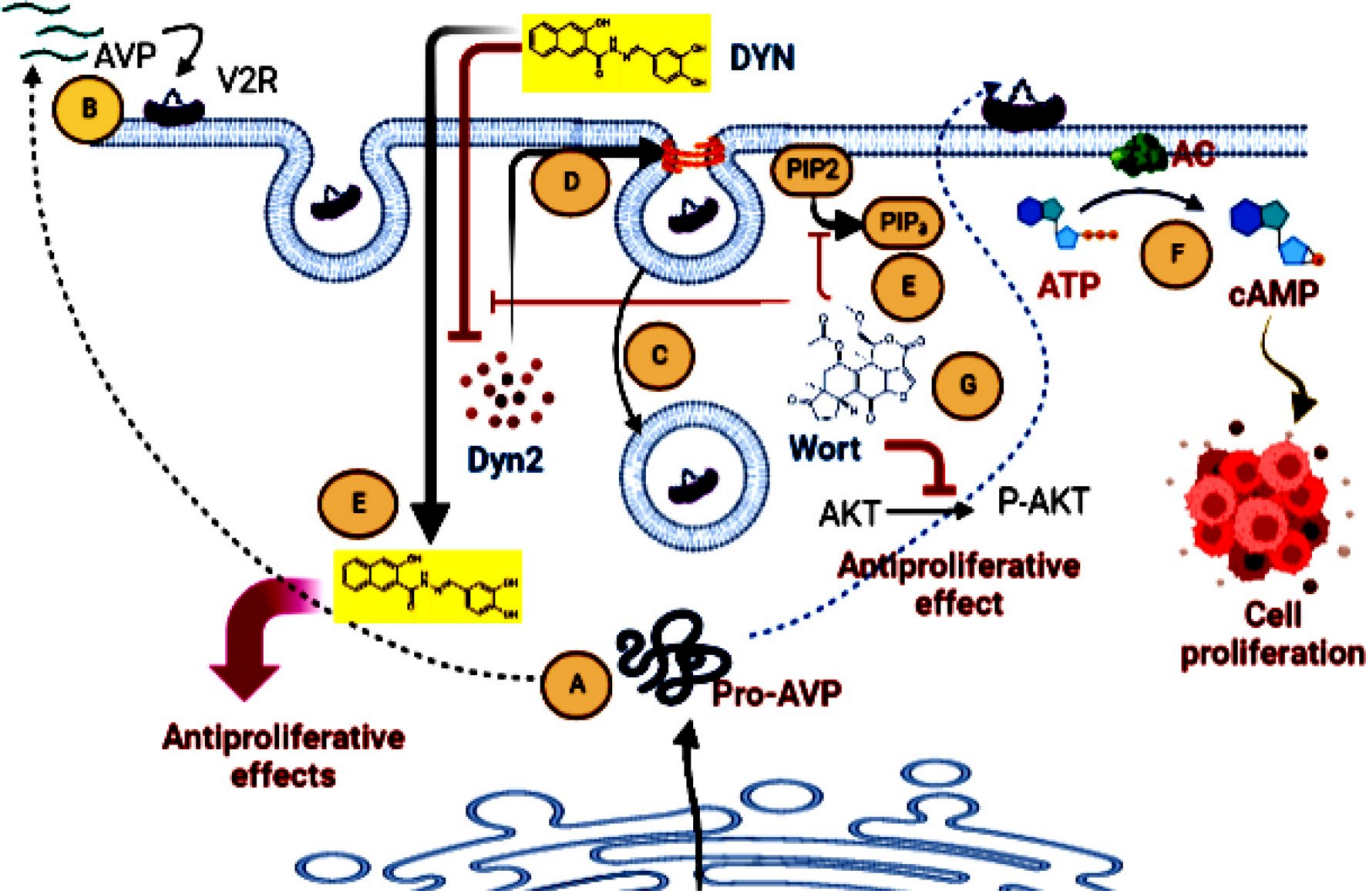

## Introduction

Breast cancer is the most prevalent metastatic tumor and the main cause of cancer-related death among women all over the world, where its curability remains a challenging task till now [1]. Long-term exposure to high level of steroidal hormones plays an integral role in the incidence of breast cancer, whereas the role of non-steroidal hormone was inadequately investigated. AVP, for example, is constitutively synthesized in the hypothalamus and functions mainly on kidney’s collecting duct cells. The signaling effect of AVP is mediated by G-protein coupled receptors (GPCRs) including vasopressin type 1 receptors (V1aR, V1bR also called V3R) and vasopressin type 2 receptor (V2R) [2, 3]. AVP and its receptors, however, are ectopically expressed in several cancers, including breast cancer. Breast cancer cell lines, like MCF-7, Skbr3, BT474, ZR75 and Mcf10a, in addition to human breast cancer tissues, were found to abnormally express AVP [4]. Moreover, earlier studies showed that MCF-7 cells express mRNAs of all isoforms of the AVP receptors [5]. This makes breast cancer cells responsive to AVP as an autocrine growth factor. Beyond its classical functions in regulating water and solute transport in renal cells, AVP/V2R downstream signaling may involve a range of other cellular functions [6]. Expression of AVP in some cancers leads to the production of both normal and abnormal forms of tumor AVP mRNA and proteins [7] and triggers the growth of cell lines like breast, small cell lung cancer [8, 9], and mamary tumor growth in vivo [10]. In contrast, some investigations showed that AVP had no stimulatory effect on growth of small cell lung cancer or adreno-cortical tumor [11]. Other reports demonstrated that AVP has dose-dependent contradicting effects [12]. Regardless the receptor isoform it binds with, AVP may undergo clathrin-mediated endocytosis (CME) that involves several cellular endocytic proteins including Dynamin 2 (Dyn2), a member of large-guanosine 5’-triphosphatase (GTPase) family. Structurally, Dyn2 is multimodular protein composed of five conserved domains, including the large N-terminal GTPase domain (also, called G-domain) [13, 14]. In CME, soluble Dyn2 monomers are recruited to the neck of the nascent liganded receptor-containing clathrin coated vesicles (CCVs) forming a helical oligomer that catalyzes CCVs fission from plasma membrane [15, 51]. In 2006, 3-hydroxynaphthalene-2-carboxylic acid-(3,4-dihydroxybenzylidene)-hydrazide (commonly known as Dynasore, DYN) was introduced as the first large dynamin GTPase inhibitor, where it selectively inhibits Dyn isoforms, (Dyn1, Dyn2 and Dyn3 GTPases), without affecting small GTPases [16]. Compared to RNAi-mediated inhibition of genes encoding Dyn2, Dynasore also, reduces labile cholesterol in the plasma membrane and disrupts lipid raft organization in a dynamin-independent manner [17], that may enhance its role as an endocytic inhibitor. The direct involvement of Dyn2 in cancer development (reviewed by [21]), nominated him as a promising anticancer target. In the same context, some reports revealed that invasive cancer cells requires Dyn2 for the internalization of several proteins involved in cancer cell motility and invasiveness by the endocytosis process [18]. Other studies have reported that Dyn2 may act as a facilitator of focal adhesion turnover during cell migration and invasion [19], and vascular endothelial growth factor (VEGF)-mediated angiogenesis [20, 21]. Collectively, these observations suggest the key role of Dyn2 in the progression of luminal A breast cancer towards invasiveness and metastasis. In this regard, the combined effect of AVP stimulation and Dyn2 inhibition was not adequately exploded. Moreover, it is not clear how the DYN-mediated inhibition of Dyn2 will modulate AVP-stimulated breast cancer cells proliferation, and migration. Thus, this work is designed to explore the anti-proliferative and antimetastasis effects of Dynasore in a luminal A breast cancer cells, which was prestimulated with exogenous AVP. Also, the associated involvement of phosphatidylinositol 3-kinase (PI3K) pathway will be investigated.

## Materials and methods

### Key reagents

Dynasore (3-Hydroxy-[(3,4-dihydroxyphenyl) methylene] hydrazide 2-naphthalenecarboxylic acid; Cat No., D826508), Arginine Vasopressin acetic acid salt (AVP) (Cat No., V991535) and Wortmannin, (Cat No. W499400) were purchased from Toronto Research Chemicals, Canada. Cell culture reagents (Dulbecco’s modified Eagle’s Minimal Essential Medium (DMEM) with L-glutamine, penicillin/streptomycin, fetal bovine serum (FBS) and Trypsin/EDTA) were from Lonza Pharma & Biotech, Basel, Switzerland. Total and P-Akt monoclonal antibodies were from Cell Signaling Technologies (Ma, USA).

### Cell culture and treatment

MCF-7 cells were purchased from VACSERA, Cairo, Egypt. Cells were seeded and maintained in DMEM supplemented with 10% heat-inactivated FBS and 1% Penicillin/Streptomycin. Cells were incubated in 95% humidified air and 5% CO_2_ at 37 °C. Initially, cells were seeded with low cell density and then subcultured with particular densities in T75, T25 tissue culture flasks, 6-well plates, or 96-well plates, according to the experimental settings. Dynasore (dissolved in DMSO) was used to inhibit DYN2. The V2R receptor was stimulated by treatment of cells with 100 nM AVP and PI3K/Akt/mTOR pathways were inhibited by 100 nM Wortmannin for 24 h.

### Viability assay

Cell metabolic activity was determined using a MTT assay. Briefly, cells were cultured in 96-well plates at a density of 2×10^4^ cells/well. After overnight incubation, for cells attachment, DMEM media was replaced with fresh media containing different concentrations of Dyn and incubated at 37 °C in 5% CO_2_ for 24 h. Cells were then labeled with 20 μl of MTT solution (5 mg/mL in PBS) per well. After 5 min shaking, cells were incubated in the dark for 4 h. The medium was then removed, dimethyl sulfoxide (DMSO) was added to dissolve the formazan, and then wells absorbance was measured at 546 nM.

### Flow cytometry for apoptosis, autophagy and cell cycle assessments

Apoptosis assay was performed using Annexin-V FITC kit (Miltenyi Biotec, CA, USA) following the manufacturer’s instructions. Briefly, subconfluent cells treated with AVP, DYN+AVP, Wort, DYN+Wort, and AVP/Wort/DYN were detached by trypsinization and then centrifuged at 1000 rpm for 5 min. The cells pellet was resuspended in 1 ml PBS and incubated with 0.25 μg/ml Annexin-V in 1X binding buffer for 15 min, followed by two washes with Wash Buffer. Cells were resuspended again in a binding buffer containing 0.5 μg/ml Propidium Iodide (PI) and then subjected to a flow cytometer (BC, Novus). The data were analyzed by Kaluza software. In parallel, autophagy flux was determined by measuring the abundance of LC3II protein by fluorescent antibody labeling of the microtubule-associated protein using Rabbit anti-Homo sapiens MAP1LC3B Polyclonal antibody (MAP1LC3B Antibody, FITC conjugated) (CUSABIO, USA). For cell cycle analysis, cells were treated with AVP, Wort, AVP+DYN, Wort+DYN, AVP+Wort+DYN or DMSO. Cells were harvested, washed twice with PBS then fixed with 70% ethanol (in PBS, v/v). After incubation, at 4 °C for at least 2h, cells were washed with PBS and stained with PBS containing PI (50 μg/ml, Triton ×-100 and RNaseA) for 30 min at room temperature in a dark place. Cells suspension was filtered then analyzed for cell cycle by Accuri C6 flow cytometer (Becton Dickinson, Sunnyvale, CA, USA).

### Akt and P-Akt expression

The ready Prep^TM^ protein extraction kit (Bio-Rad Inc., Catalog No., 163-2086) was used to extract total protein according to the manufacturer’s instructions. Bradford Protein Assay Kit (Bio Basic Inc., Markham Ontario, Canada) was utilized to determine protein concentration following the manufacturer’s instructions. For blotting, 20 μg protein was mixed with an equal volume of 2× Laemmli sample buffer (4% SDS, 10% 2-mercaptoethanol, 20% glycerol, 0.004% bromophenol blue, and 0.125M Tris HCl, pH 6.8), where the mixture was boiled at 95 °C for 5 min before loading on polyacrylamide gel. The blot was run, followed by membrane blocking at room temperature for 1 h. Primary antibodies of total and phosphorylated Akt were diluted in TBST and incubated overnight with the membranes at 4 °C. The blot was rinsed 3–5 times for 5 min with TBST and then incubated with the HRP-conjugated secondary antibody (Goat anti-rabbit IgG- HRP-1mg Goat mab-Novus Biologicals) for 1 h at room temperature. After another wash with TBST, the chemiluminescent substrate (Clarity TM Western ECL substrate Bio-Rad cat#170-5060) was applied and the signals were captured using a CCD camera-based imager. Image analysis software (Image J) was used to read total-Akt and P-Akt band intensities.

### Cells invasion assay

MCF-7 cells were cultured in 6 well plates and left to grow up to 70% confluence, after which they were treated as shown above. By the end of the treatment period, cells were trypsinized, washed with PBS, and resuspended in serum-free DMEM. Inserts (8 μm pore size, BD Biosciences, St Louis, USA) were mounted onto the top of 6 well plates, where 100 μl serum-free medium and 200 μl of treated cells (2.5×10^5^ cell/ml) in serum-free medium were added to the upper chamber. In the lower chamber, 750 μl of 15% serum-containing medium was added, and then the treated cells containing inserts were placed onto the lower champers. The plates were kept at 37 °C for about 18 h, after which the media were decanted and the inserts were washed twice with PBS. Cells were fixed with 3.7% formaldehyde (in PBS) for 2 min at room temperature. After decanting the formaldehyde, cells were washed twice with PBS and permeabilized with 100% methanol for 20 min at room temperature. Methanol was decanted and cells were washed twice with PBS, stained with 300 μl Giemsa stain, covered with tin foil, and then incubated for 15 min at room temperature. The stain was removed and cells were washed twice with PBS. Non migrated cells were scraped off with a cotton swab, and then the membrane was photographed under the light microscope, where the average number of migrated (transmitted) cells was counted using Image J software.

### RNA isolation, cDNA synthesis, and expression analysis

Quantitative real-time PCR was employed to determine the fold expression of Beclin-1, MDR,1 ProAVP, Bax, and Caspase-3 genes at mRNA levels using Qiagen Rotor-Gene QPCR Cycler 5 Plex. Initially, total RNA was purified using a GeneJET RNA purification kit, (ThermoFisher Scientific, USA). After quantitation and quality assessment, 200 ng RNA was used as a template for cDNA synthesis, using SensiFAST™ cDNA Synthesis Kit (Bioline Inc, USA) following the manufacturer’s protocol. For real-time PCR quantitation, 50 ng/μl (2 μl) of cDNA was used as a template in 20 μl thermal cycling reactions containing 50 nmol/μl (2 μl) of the genes-specific primers (Table 1), the ready-to-use master mix of fluorescent dye SYBR green 1 and HotStar *Taq* DNA polymerase. Reactions were subjected to a thermal cycler program consisting of a single denaturation step followed by 45 cycles (each consisted of a denaturation step at 94 °C for 5 s, annealing at 58, 56.8, and 57.9 °C (for Pro-AVP, Bax, and Caspase3, respectively) and an extension step at 72 °C for 20 sec. Reactions were terminated with a single step at 99 °C to produce melt curves. In parallel, the expression of the GAPDH gene was used as an internal control to determine the relative fold expression of the targeted genes. The critical threshold (C_t_) of target genes was normalized with quantities (C_t_) of GAPDH using the 2^−ΔΔCt^.

**Table 1:** Sequence of primers used in the expression analysis of apoptotic genes and Pro-AVP

Gene		Sequence (5′–3′)
ProAVP	For	5′-CTTCTCCTCCGCGTGCT-3′
	Rev	5′-CGTCCAGCTGCGTGGCGTTGC-3′
BAX	For	5′-AAGCTGAGCGAGTGTCTC-3′
	Rev	5′-TCCCGCCACAAAGATGGT-3′
Caspase3	For	5′-TTTGTTTGTGTGCTTCTGAGCC-3′
	Rev	5′-ATTCTGTTGCCACCTTTCGG-3′

*For Forward (sense), Rev Reverse (antisense)*

### Statistical data analysis

Data analysis was performed using the SPSS13.0 software package. All cell culture work was performed in triplicates. Apoptosis and the autophagy markers were measured and displayed in histograms as a percent of the control and represented as the mean of 3 runs ± standard deviation. *P* values less than 0.5 indicate significant differences.

## Results

**Fig. 1:**
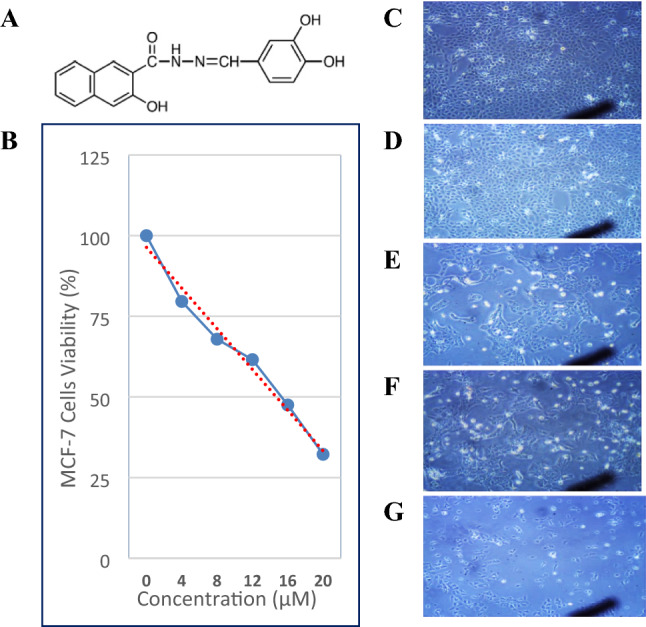
Cytotoxic effect of Dynamin inhibition. Dynasore (**A**) induced a concentration-dependent cytotoxic effect in breast cancer cells. MCF-7 cells were incubated for 24 h with varying concentrations of Dynasore and cells metabolic activity was determined by MTT assay. Data are expressed as means ± SD of multiple experimental replicates (*n* = 5) (**B**). C–H are representative phase contrast photomicrographs of cells treated with AVP (**C**), DYN after cells stimulation with AVP (**D**), Wort (**E**), DYN in combination with Wort (**F**) or DYN in combination with Wort and AVP (**G**). Apoptotic morphological abnormalities including cell shrinking, rounding and detachment (magnification of 400×)

**Fig. 2:**
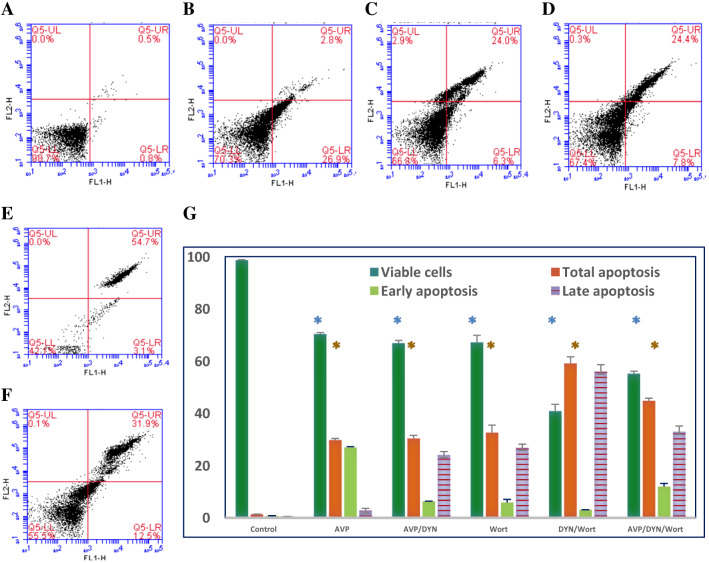
Apoptotic effect of dynamin 2 inhibition in invasive breast cancer cells. Plots (**A**) through (F) show Annexin V-FITC and PI-stained MCF 7 cells untreated (**A**), treated with AVP (**B**), DYN after stimulation with AVP (**C**), Wort (**D**), DYN in combination with Wort (**E**) or prestimulated with AVP then treated with a combination of Wort and AVP (**F**). In each scatter plot, the lower left quadrant, the upper left quadrant, the lower right quadrant and upper right quadrant represent the percent of viable cells, dead cells, early apoptotic cells and late apoptotic cells, respectively. Apoptosis was observed in DYN treated cells and more apoptosis developed in presence of AVP or Wort combined with DYN treatment (**G**). Results are presented as mean ± SD. *DYN* Dynasore; *Wort* Wortmannin, *AVP* arginine vasopressin; (*) refers to *P* < 0.001 and indicating highly significant viability or total apoptosis relative to DMSO-treated cells

**Fig. 3:**
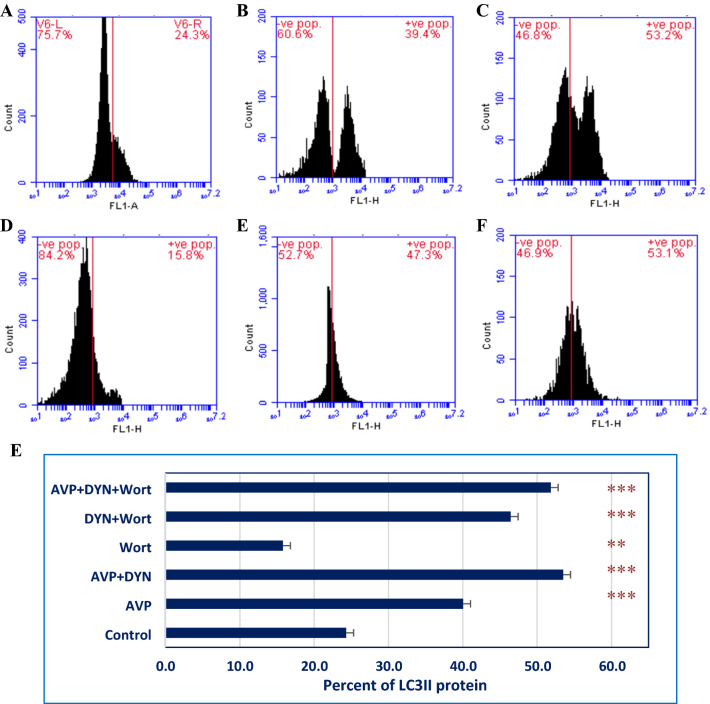
AVP stimulates autophagy and enhances the autophagy effect of Dynamin inhibition, promotes cytotoxic PI3K-independent autophagy in breast cancer cells. McF-7 cells were seeded with an initial cell density 4×10^4^ grown in nutrient-rich conditions and left untreated (**A**), transiently treated with AVP (**B**), DYN after cells stimulation with AVP (**C**), Wort (**D**), DYN in combination with Wort (**E**) or DYN in combination with Wort and AVP (**F**). Following treatments, the expression of the autophagy marker (LC3II protein) was determined by flow cytometry. The autophagy was significantly induced in Dynamin inhibited cells and reduced in Wort-treated cells. Dyn2 inhibition induced significant increase in LC3II even in cells in which PI3K was inhibited by Wort. Data are presented as mean ± SD (**G**). *DYN* Dynasore; *Wort* Wortmannin, *AVP* arginine vasopressin; (_***_) and (**): refer to extremely significant (*P* < 0.001) or highly significant differences (*P* < 0.01) between the indicated group compared to DMSO-treated cells

**Fig. 4:**
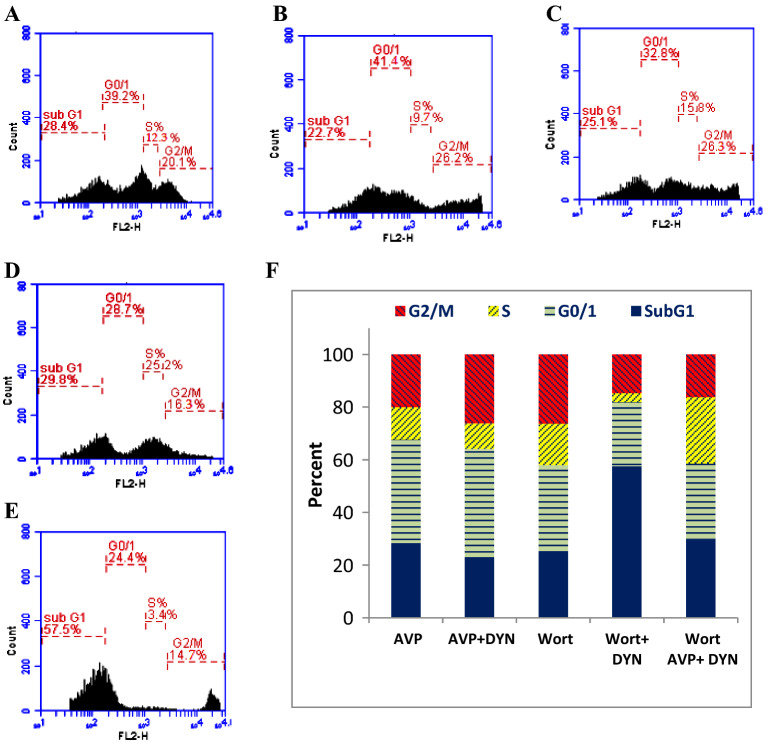
Dynamin inhibition induces cell cycle arrest in G0/G1 phase in breast cancer cells. MCF-7 were treated with AVP (**C**), DYN after cells stimulation with AVP (D), Wort (**E**), DYN in combination with Wort (**F**) or DYN in combination with Wort and AVP (**G**). After cell fixation, they were PI-stained and analyzed for cell cycle by flow cytometry. **A**–**E** are representative scatter plot of cell cycle analysis and (**F**) is bar graph of cell fractions distributed in different phases. DYN and AVP arrested cells in G0/G1 phase

In order to assess cell viability and determine the IC_50_ of Dynasore (DYN), the changes in the metabolic activity of cells were measured by MTT assay. Dynasore treatment decreased the viability of MCF-7 cells in a concentration-dependent manner, with IC_50_ value 8.6 μM (Fig 1). Also, cells treated with DYN, in the absence or presence of AVP stimulation or Wort, showed apoptotic morphological changes including cell shrinking, rounding, and floating. Representative images of treated cells and the associated changes in cell viability are shown in Fig. 1C-G. As indicated by the Annexin-VFITC/PI staining, the percent of viable cells decreased from 98.7% in DMSO-treated cells to 70.3±0.7%, 66.8±1.2%, 67.4±1.1%, 67.2±2.7%, 40.8±2.6%, and 55.2.8±1.0%, in cells treated with AVP, AVP+DYN, Wort, DYN+Wort, and AVP+DYN+Wort, respectively. Also, flow cytometry analysis revealed that cells prestimulated with exogenous AVP or treated with DYN after AVP stimulation showed combined early and late apoptosis in 29.7±0.7% and 30.3±1.2%, however the first revealed higher early apoptosis (26.9%) than AVP/DYN treated cells (6.3%). Cotreatment of cells with DYN and Wort in cells unstimulated with AVP-induced apoptosis in 59.1±2.6%., whereas a relatively lower apoptosis (44.7+2.6%) was detected in AVP-prestimulated cells. All the observed viability changes and the apoptotic effects of different treatments were significant (*P*<0.001) compared to DMSO-treated cells (Fig. 2). To investigate whether Dyn2 inhibition was associated with autophagy, fluorescent monoclonal antibody labeling of the microtubule-associated protein, LC3II was estimated and compared to its basal level measured in DMSO-treated cells (24.3%). The levels of LC3II, when cells exposed to AVP alone, AVP+DYN, Wort, DYN+Wort, and AVP/DYN/Wort were 40.03±3.0%, 53.5±0.4%, 15.8±0.8%, 46.4±1.4%, and 51.8±2.2%, respectively, compared to its basal level (24.3%) detected in control cells (Fig. 3). Cell cycle analysis revealed that the highest SubG1 fraction associated with the lowest percent of cells in S phase were observed in cells dually treated with DYN+Wort (Fig. 4)

**Fig. 5:**
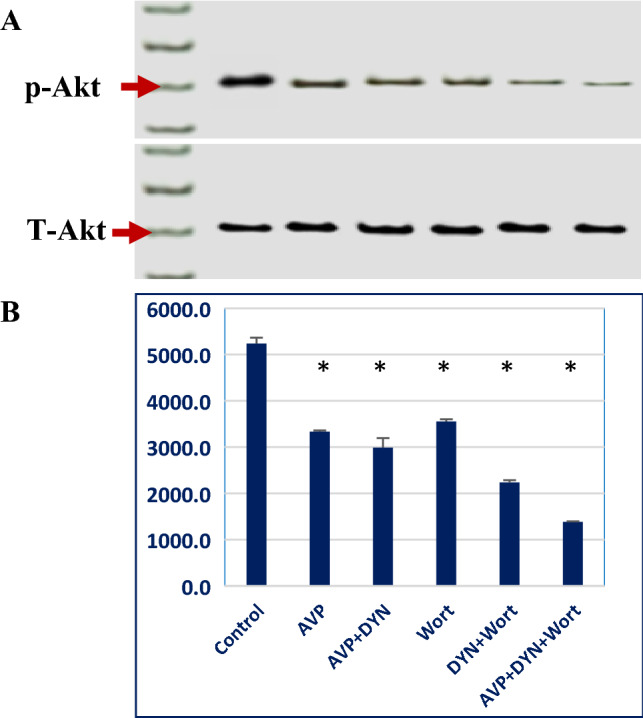
Expression of total Akt and phosphorylated Akt (P-Akt) proteins at (Ser473) in breast cancer cells stimulated with AVP and treated with Dynamin 2 and/or PI3/AKT inhibitors by Western blot analysis. Top panel (**A**) MCF-7 cells in which V2R receptor was prestimulated with AVP or PI3K pathway was inhibited by Wort in combination with the dynamins inhibitor (DYN). Cell lysate was analyzed using Akt or P-Akt monoclonal antibodies. Bottom (**B**) represents the mean of bands intensities of the quantification of Total Akt or P-Akt (Ser 473) as means ± SD of data from independent experiments (*) refers to *P*<0.001 and indicates significant changes in the corresponding cells compared to DMSO-treated cells; *DYN* Dynasore; *Wort* Wortmannin, *AVP* arginine vasopressin

Next, the involvement in PI3/AKT/mTOR pathway modulation was investigated, where cells were stimulated with AVP then incubated with DYN or/and Wort that selectively inhibits PI3K/mTOR pathway at PIP2–PIP3 transition reaction. The immunoblotting results demonstrated that AVP alone or in cells prestimulated with AVP then treated with DYN, significantly decreased the level of phosphorylated AKT (P-AKT) compared to the corresponding level in DMSO-treated cells and as similar as in Wort-treated cells. Also, DYN demonstrated a synergistic effect with Wort, where a lower P-AKT expression was observed in cells cotreated with DYN and Wort. The least level of P-AKT was obtained in cells treated with DYN in presence of both AVP and Wort (Fig. 5).

**Fig. 6:**
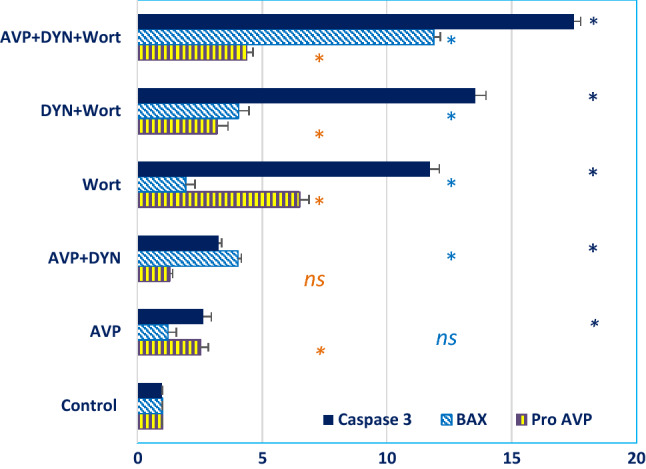
Relative expression of Bax, Caspase-3 and Pro-AVP genes in dynamin 2 inhibited breast cancer cells. MCF-7 cells were treated with DYN alone, after their prestimulation with AVP, or cotreated with Wort. Cells mRNA was isolated, reverse transcribed, and the cDNA was used as a template in qRT-PCR to determine the relative expression. Dynamin inhibition was associated with up-regulation of apoptosis-related genes and Pro-AVP expression (*) indicates significant difference between the indicated cells versus control cells; (ns) indicates non-significant

**Fig. 7:**
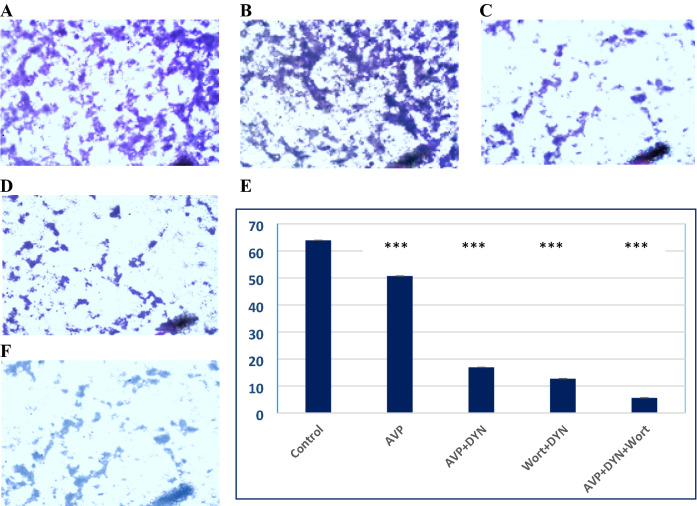
AVP reduced the invasion potential of breast cancer cells assessed by transwell assay. Untreated MCF-7 cells (**A**), cells stimulated with AVP (**B**), AVP + DYN (**C**), DYN + Wort (**E**), or AVP + DYN + Wort (**F**), respectively. After treatments, cells were resuspended in serum-free media, added to the upper chamber (inserts), kept at 37 °C for 18 h, fixed with formaldehyde, permeabilized by methanol, washed and then stained with Giemsa stain. Images were captured under inverted microscope and analyzed by Image J. (**E**) represents the relative changes in the mean area, representing the migrated cells, where treatments significantly (*P* < 0.001) reduced cell invasiveness

Expression analysis at the mRNA level included the expression of 2 apoptosis-related genes (Bax and Caspase-3) and the endogenous AVP (Pro-AVP). The results indicated that Dynasore led to the upregulation of both Bax and Caspase-3, especially in cells prestimulated with AVP. The expression levels of both apoptotic genes were progressively increased when cells treated with Wort, DYN+Wort and AVP+DYN+Wort. Moreover, the expression of Pro-AVP mRNA was variably expressed in MCF-7 cells and the expression was enhanced in cells dually treated with Dyn+Wort in presence of absence of AVP prestimulation (Fig. 6). To monitor the impact of AVP stimulation and Dyn2 inhibition on breast cancer cells invasion, transwell assay was performed after cells being treated with AVP, DYN+AVP, DYN+Wort, and AVP/Wort/DYN. As Fig. 7 shows, both AVP reduced cells invasion and enhanced the effect of Dynamin 2 and PI3k/Akt inhibition as well.

## Discussion

The hormonal involvement in breast cancer is well-reported in the incidence, metastasis and treatment as well [22]. The incidence, in particular, is modulated through the exposure of cells to high levels of steroidal hormones [23]. Also, breast cancer cells ectopically synthesize the neuropeptide AVP as a prohormone, where the autocrine responsiveness of cells is granted by the associated expression of AVP receptors. The antiapoptotic effect of AVP was reported in several cancers (as summarized in table 2), including breast cancer [4]. Based on semilar studies, the effect of AVP on tumor growth is dose-dependent [8, 9, 24]. In this work, the experimental design included cells affected by the autocrine effect of AVP or cells affected by both the endogenous AVP in addition to 100 nM AVP. The results revealed that MCF-7 cells expressed pro-AVP mRNA, where the expression was variablly modulated in cells treated with exogenous AVP and/or PI3K/Akt inhibition. Exogenous AVP, for instance, induced about 2-fold increase in the expression of pro-AVP mRNA, meanwhile higher expression level (6-fold) was observed in Wort-treated cells. In contrast to some reports, we found that apoptosis was developed in about 1/3rd of cells prestimulated with AVP. This may be explained by the AVP-mediated hyperactivation of ERK1/2, that triggers cells apoptois after its nuclear localization [25]. Alternatively, AVP-induced apoptosis may occur through the mitochondrial apoptotic pathway, where at least 2-fold increament was observed in the expression of both apoptosis-related genes (Bax and Caspas-3), the observation previouly reported [12, 26]. Based upon previous observations, it seems that AVP derived antiapoptiotic or apoptotic effects are dose-dependent. The stimulatory or inhibitory effects in MCF-7 cells, for example were associated with lower (0.01–1nM) and higher doses (above 10nM), respectively. Moreover, growth-related effects are modulated according to the receptor isoform [12, 26].

**Table 2:** Apoptotic or antiapoptotic potential of arginine vasopressin

Cancer/cells	Receptor (subtype)	Effect	References
Renal collecting duct cells (mpkCCD)	V2R	Antiapoptotic	[43]
Glomerular mesangial cells	V1a	Antiapoptotic	[44]
Neuronal cell line H32	V1a, V1b & V2R	Antiapoptotic	[45]
Hippocampal Neurones	V2R	Apoptotic	[46]
Renal cell carcinoma (RCC)	V2R	Antiapoptotic	[12]
Breast cancer (MCF7 & Skbr3 cells)	V1,V2	Apoptotic	[47–49]
Castration-resistant prostate cancer (CRPC)	V1R	Apoptotic	[50]

Although the detailed scenario of AVP downstream signaling was not precisly explored, the liganeded AVP receptors may intiate 2nd messenger-mediated downstream effect or it may undergo receptor-mediated endocytosis (RME), similar to other peptide hormones [27]. The internelization of the liganed-receptor complex involves several endocytosis-related proteins including Dyn2. In addition to its integral role in RME, some investigations demosnstrated that Dyn2 overexpression is associated with more aggressive tumor phenotype in breast cancer patients, indicating its role as an indicator of disease progression and aggressiveness [28]. Herein, Dyn2 was selectively inhibited by Dynasore (DYN), that interacts with the G domain of Dyn2 to prohibit its internsic GTPase activity. This may restrict its oligomerization and the subsequent its utelization in vesicle fission and endosome formation. Compared to other dynamins inhibitors (like Dynole 34–2 and Mdivi-1), DYN is known to selectively inhibit Dyn2, predomenently expressed in all tissues, including breast cells, in addition to its inhibitory effect on the mitochondrial dynamin-related protein (Drp-1) [16]. More importantly, DYN does not affect small GTPases like *Ras, Rho, Rab* families [29]. In previous work, we demonstrated that the inhibition of the interaction between *β*-arrestin, an endocytic accessory protein, and AP2 adaptor protein, demeinshed the viability of triple negative breast cancer (TNBC) cells [30]. In a similar manner, the aim here was to scale the associated non-endocytic related side effects of Dyn2 inhibition on breast cancer cells viability and migration potential, especially when these cells are stimulated with exogenous AVP. The involvement of Dyn2 in the biphasic insulin secretion, from β-cells, and glucose homeostasis [31] may explain the progressive and concentration dependent decline of cells metabolic activity in DYN-treated cells. Also, DYN-mediated inhibition of Dyn2 accelerated the development of late apoptosis in cells prestimulated with AVP, where 24% of cells were in the late apoptosis compared to 2.7% of cells transiently exposed to AVP. Moreover, Dyn2 inhibition, synergistically, enhanced the apoptotic effect of Wort-mediated PI3K/Akt inhibition, especially in AVP unstimulated cells (about twofold increase). The role of Wort is explained by its selective inhibition of PIP2 phosphorylation and the subsequent inhibition of PI3K/Akt/mTOR pathway [32]. Moreover, the indirect effect of Wort may occur through the inhibition of Dyn2 recruitment to the cell membrane mediated through the inhibition of synthesis of PIP2 and PIP3 production [33]. During these events, more soluble Dyn2 is porn to the inhibitory effect of DYN. In contrast, the massive RME-mediated consumption of Dyn2 in AVP-stimulated cells, may explain the relative decrease of DYN-induced apoptosis in cells exposed to AVP/DYN/Wort. This makes the availability of soluble Dyn2 as a limiting factor in DYN-mediated cytotoxic effect.

In addition to the apoptosis, the cytotoxic autophagy effect of AVP with or without DYN was evidenced by the at least 2-fold increase in the autophagy marker (LC3II), the observation previously reported in endothelial cells [34]. Both the direct and indirect roles of Dyn 2 in regulating autophagy were previously reported. The direct effect takes place through autophagosome formation and autophagic lysosome reformation through its excision activity. The indirect regulatory effect may take place through the regulation of lysosomal function [35]. In contrast, Wort led to downregulated autophagy, the finding we and others previously reported in breast cancer MDA MB232 cells [30], in brain cancer [36] and colorectal cancer [37]. Also, a significant decrease in cell migration was resulted in cells treated with DYN. Similar effects were observed in other cancers like colon, cervix, lung, pancreas, brain, and prostate [38, 39]. Also, several studies reported that inhibition of Dyn2 was associated with prevention of rapid turnover of adhesion molecules, which are required for cell migration [40–42]. In this regard, Dyn2 plays important role in the dynamin-dependent endocytosis pathways, like clathrin-mediated endocytosis (CME) and caveole-mediated endocytosis CAE, that faciliate the entrance of protein responsible of cancer cell motility and invasion [21]. In the same context, PI3K/Akt/mTOR pathway is frequently deregulated in cancer cells. In breast cancer, it plays an essential role in tumor proliferation and enhances an endocrine resistance. AVP transient treatment, reduced Akt phosphorylation to as similar as the PI3K/Akt inhibitor. This agrees with the observed changes in cell death and the reduction of the metastasis potentials of breast cancer cells.

In summary, the results suggest the antiproliferative, the autophagic and the antimetastasis effects of high (100 nM) does of AVP as similar as the PI3K inhibitor. Also, the combined effect of AVP and DYN demonstrated less apoptotic effect compared to DYN and Wort. More investigations are required to demonstrate the role of different receptors isoforms and the involved downstream signaling.

## Data Availability

Data sharing is not applicable to this article.

## Abbreviations

AVP: Arginine vasopressin
RME: Receptor mediated endocytosis
Dyn2: Dynamine2
DYN: Dynasore
PIP2: Phosphatidylinositol 4,5-bisphosphate
PIP3: Phosphatidylinositol (3,4,5)-trisphosphate
